# Glucose Tolerance during Pulmonary Exacerbations in Children with Cystic Fibrosis

**DOI:** 10.1371/journal.pone.0044844

**Published:** 2012-09-13

**Authors:** John Widger, Mark R. Oliver, Michele O’Connell, Fergus J. Cameron, Sarath Ranganathan, Phil J. Robinson

**Affiliations:** 1 Department of Respiratory Medicine, The Royal Children’s Hospital, Melbourne, Victoria, Australia; 2 Department of Gastroenterology, The Royal Children’s Hospital, Melbourne, Victoria, Australia; 3 Department of Endocrinology, The Royal Children’s Hospital, Melbourne, Victoria, Australia; 4 The University of Melbourne, Melbourne, Victoria, Australia; 5 Murdoch Children’s Research Institute, Melbourne, Victoria, Australia; Johns Hopkins School of Medicine, United States of America

## Abstract

**Background:**

Patients with Cystic Fibrosis (CF) are relatively insulinopenic and are at risk of diabetes, especially during times of stress. There is a paucity of data in the literature describing glucose tolerance during CF pulmonary exacerbations. We hypothesised that glucose tolerance would be worse during pulmonary exacerbations in children with CF than during clinical stability.

**Methods:**

Patients with CF, 10 years or older, admitted with a pulmonary exacerbation underwent an OGTT within 48 hours of admission. A repeat OGTT was performed 4 to 6 weeks post discharge when the patients were well.

**Results:**

Nine patients completed the study. Four patients were found to have normal glucose tolerance, 3 with impaired and 2 with CF related diabetes during the exacerbation. Mean change in 2-hour glucose was 1.1 mmol (SD = 0.77). At the follow up OGTT, 8 of 9 (89%) remained within their respective glucose tolerance status groupings.

**Conclusion:**

The findings of this study show that there is little difference in glucose tolerance during CF exacerbations compared to clinical stability in the majority of patients.

## Introduction

Cystic Fibrosis (CF) is the most common inherited life limiting condition in Caucasians [1]. Patients with CF and pancreatic insufficiency are relatively insulinopenic and are at risk of impaired glucose tolerance (IGT) and CF related diabetes (CFRD) [2]. It is well established that the presence of these co morbidities has a significant impact on morbidity and mortality in CF [3]. Recent international consensus guidelines recommend that all CF patients should be tested for IGT and CFRD yearly with an oral glucose tolerance test (OGTT) from 10 years of age [4].

CF pulmonary exacerbations are episodes of acute deterioration, usually brought on by infection and characterised by an increase in respiratory symptoms [5]. They are associated with decreased quality of life and increased mortality, hospitalization and healthcare costs. In the non-CF population, stress hyperglycaemia can occur during acute illness and is associated with adverse outcomes in children [6]. It is widely believed that stress hyperglycaemia occurs during CF pulmonary exacerbations and may resolve following resolution of the exacerbation [4]. However, evidence for this is lacking with only one small study having examined glucose tolerance during pulmonary exacerbations. Sc and colleagues performed both intravenous glucose tolerance tests (IVGTTs) and OGTTs on 8 CF patients previously known to have normal glucose tolerance (NGT) [7]. All but one patient exhibited diabetic glucose tolerance on OGTT during pulmonary exacerbation. Furthermore, patients returned to NGT within 4 weeks of discharge. These findings suggest that CF patients may benefit from insulin treatment during exacerbations however such results require confirmation before the development of specific randomized controlled trials.

We hypothesised that glucose tolerance deteriorates during pulmonary exacerbations in children with CF. The primary aim of this study was to determine whether glucose tolerance status, as measured by an OGTT, is altered during a pulmonary exacerbation compared to when patients are clinically stable.

**Table 1:** Baseline characteristics of study subjects at recruitment.

**Number of subjects**	**11**
**Mean Age (years)(SD)**	14.5 (2.8)
**Gender**	8F/3 M
**Mean BMI z score (SD)**	0.02 (0.77)
**Mean %HbA1c (max,min)**	5.7(5.2,6.7)
**Genotype**	
** Homozygous dF508**	5 (45%)
** Heterozygous dF508**	3 (27%)
** Other**	2 (18%)
** Unknown**	1 (9%)
**Sputum Microbiology**	
*** P. aeruginosa***	5 (45%)
*** S. aureus***	2 (18%)
*** B. cepacia***	2 (18%)
*** Normal flora***	2 (18%)

## Methods

### Subjects

Subjects were attending the CF unit at the Royal Children’s Hospital (RCH), Melbourne, Australia. Diagnosis of CF was based on newborn screening (using immune reactive trypsinogen on a blood spot) that was introduced across the state of Victoria in 1989. This program detects about 95% of CF affected infants born in Victoria each year with the remainder detected following clinical presentations including meconium ileus, failure to thrive, suppurative chest disease, or with a CF sibling. Children with CF were eligible for the study if they were aged 10 years and older and required admission to hospital for treatment of a pulmonary exacerbation. Children were excluded from the study if they already had a CFRD diagnosis, were pancreatic sufficient or had commenced corticosteroids within the previous 6 weeks.

**Table 2:** Data on HbA1c, 2 hour glucose and BMI.

Patient	HbA1c* %	2hrg*mmol/l	BMI*z-score	BMIz**z - score
1	5.6	9.3	−1.2	−1.1
2	5.4	5.5	−0.42	−0.25
3	5.2	6.2	−0.09	−02
4	5.2	7	0.58	0.63
5#	6.1	12.6	−0.75	n/a
6	5.8	13.6	0.41	0.02
7	6.6	15.5	0.78	0.63
8	5.2	10.9	0.52	0.04
9	5.7	8.4	1.17	1.23
10	5.6	6.8	−1.01	−0.35
11$	6.7	11.8	0.2	n/a
Mean	5.7	9.78	−0.06	0.09

* On admission.

** At follow up.

HbA1c = Glycated Haemoglobin.

2hrg = 2 hour glucose.

BMI = Body mass index.

# Patient 5 died during admission.

$ Patient 11 was started on insulin and did not have a follow up OGTT.

### Study Design

A prospective observational study was carried out within the CF unit at the RCH Melbourne between the 1^st^ February 2010 and 30^th^ June 2011. At the time of the study there were around 300 patients (110 aged 10−19 years) attending the RCH CF clinic. The decision to admit patients for treatment of a pulmonary exacerbation was made by the treating physician. A pulmonary exacerbation was defined as having any 3 of the following; increased cough, change in volume, colour or thickness of sputum, haemoptysis, fever >38°C, increased shortness of breath, decreased appetite and/or weight loss, decreased exercise tolerance or lethargy, change in physical examination of the chest, radiographic evidence of pulmonary infection and an acute decline in forced expiratory volume in one second (FEV_1_) ≥10% from baseline. Eligible patients were then approached on admission and invited to participate. An OGTT was done within 48 hours of commencing IV antibiotics during the hospital admission and then repeated at 4 to 6 weeks post discharge when the patients had returned to baseline health. Participants also had a physical examination, standard spirometry, weight and height, HbA1c and sputum culture at enrollment. Spirometry was repeated prior to discharge.

**Figure 1:**
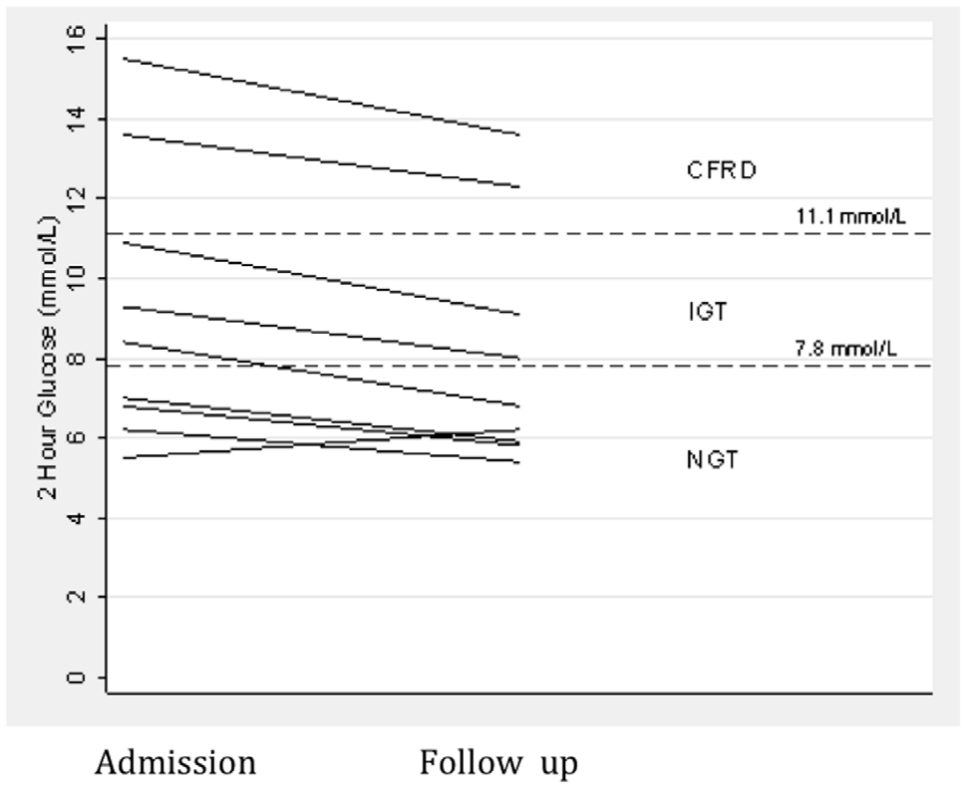
Change in 2-hour glucose measurements between admission and follow up (n = 9). Only one patient had a change in glucose tolerance status (from IGT to NGT). NGT = Normal Glucose Tolerance; IGT = Impaired Glucose Tolerance; CFRD = Cystic Fibrosis Related Diabetes.

### Ethics Statement

This study was approved by the Ethics in Human Research Committee at RCH (HREC ref: 29089A), which operates in accordance with the National Health and Medical Research Council, National Statement on Ethical Conduct in Research Involving Humans (2007). Informed consent was obtained from the parents of all participants except where participants were aged 18 years or older, in which case participants gave their own consent. All patients aged 12 years and above were asked to sign a separate participant consent form.

**Figure 2:**
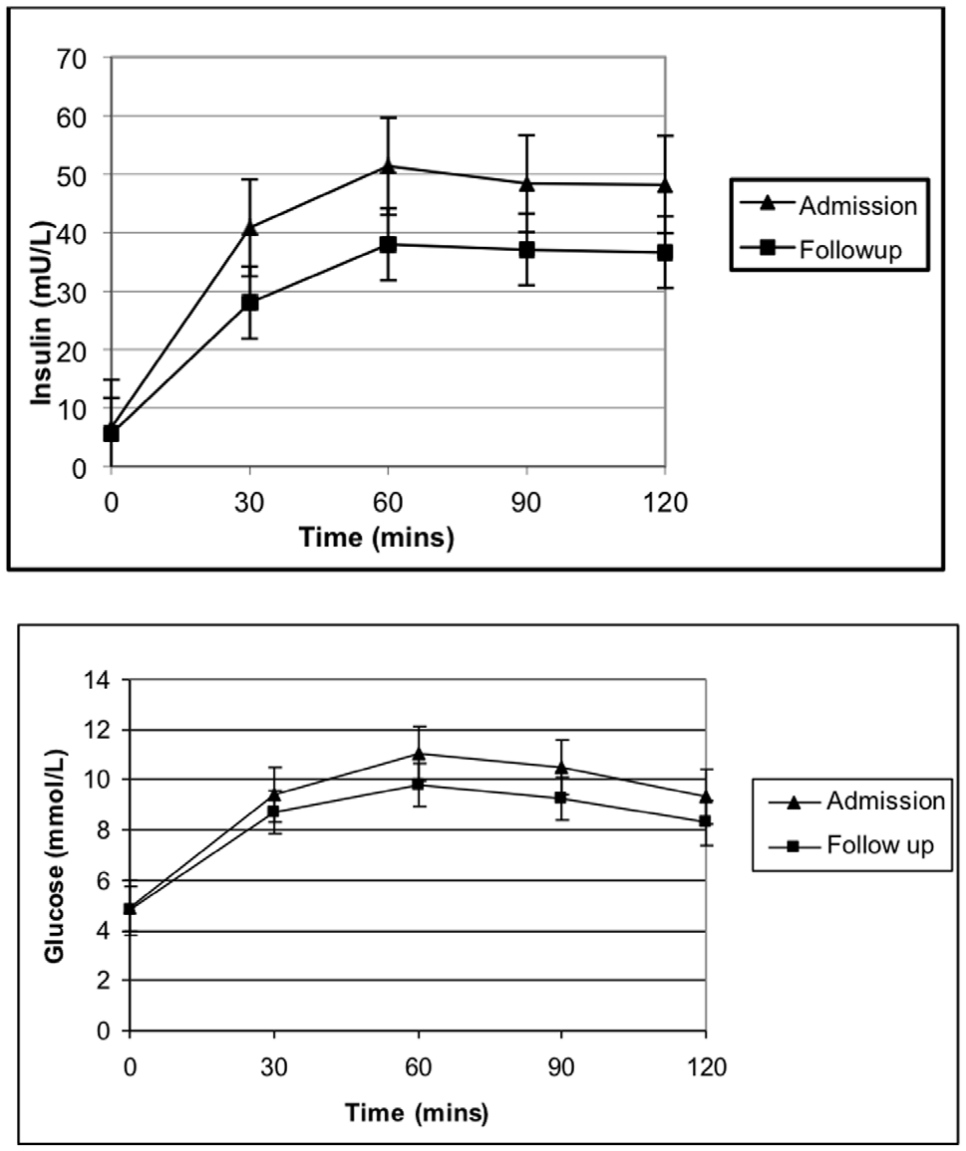
Glucose and insulin curves from 9 patients who had paired OGTTs. Mean (± SE) insulin and glucose levels are reported for each time point during OGTTS at admission and follow up. Area under the curve for glucose (p = 0.18) and insulin (p = 0.26) did not differ between admission and follow up.

### Oral Glucose Tolerance Test

The initial OGTT was performed at the bedside using the patients’ indwelling central venous or peripheral intravenous access for blood sampling. The OGTT at follow up was performed on the medical day ward. For each test, participants were asked to fast for at least 8 hours overnight. Glucose and insulin samples were taken at 0, 30, 60, 90 and 120 minutes post an oral glucose load (1.75 g/kg, max 75 g). Glucose tolerance status was defined according to WHO guidelines based on the 2-hour glucose level (<7.8 mmol/L –NGT; ≥7.8 mmol/L and <11.1 mmol – IGT; ≥11.1 mmol/L – CFRD) [8]. Insulin secretion and resistance were calculated using the Insulinogenic index **(**Insulin_30 min_- Insulin_0 mins_ (µU/ml)/Glucose_30 mins_ – Glucose_0 mins_ (mmol/L)**)** and the homeostatic model assessment (HOMA-IR = fasting Glucose (mmol/L) × fasting Insulin (µU/ml)/22.5**)** respectively.

**Figure 3:**
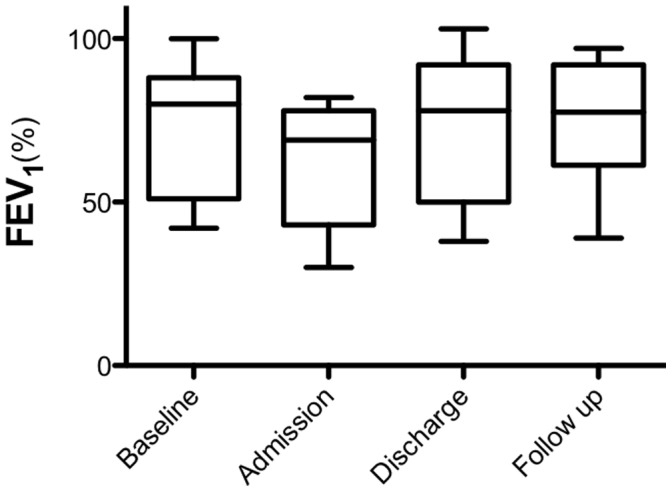
A boxplot of %FEV_1_ for each subject at each study time point. Transverse line is the mean and whiskers are maximum and minimum. There was a decline in mean %FEV_1_ from baseline to admission in all patients (mean change 10.9%, p<0.001).

### Lung Function

Spirometry was carried out according to ATS/ERS standards in an accredited lung function laboratory [9]. All study patients underwent spirometry on admission, discharge and at follow up four to six weeks post discharge. Each patient’s best lung function in the six months prior to admission was also recorded.

### Statistics

Normally distributed data were summarised using the mean and standard deviation. Non-normally distributed data were summarised using the median and interquartile range. For comparison of means for 2 sets of normally distributed continuous data with repeated measurements, a paired t-test was performed. Where data were not normal, comparison of medians was performed using a Wilcoxon rank-sum test. OGTT data were further analyzed by area under the curve (AUC) measurement using the trapezoidal method [10]. The relationship between initial 2-hour glucose and HbA1c was calculated using linear regression. Based on the study of Sc et al we calculated that a sample size of four would allow our study to detect a similar mean difference in 2-hour glucose levels (7.6 mmol/l) with a power of 80% to a significance level of 0.05. Statistical analysis was performed using Stata Version 11.0 (Stata Corporation, College Station, Texas. USA).

## Results

Twenty eligible patients met the study criteria, 11 of whom agreed to participate in the study. Nine patients completed the study, one patient died during the admission and a second patient was commenced on insulin during their admission. Complete OGTT data was available in 9 subjects. No patient received steroids during the admission. Baseline characteristics are shown in table 1. There was a female preponderance in the group. CF genotype was unavailable in 2 patients with all remaining participants having class 1–111 mutations. Table 2 shows data on OGTT results, HBA1c and BMI z-scores. There was a significant positive relationship between HbA1c and initial 2-hour glucose (r^2^ = 0.5, p = 0.01). There was no significant difference in BMI z-score between admission and follow up (p = 0.51).

### Oral Glucose Tolerance Test

Following testing on admission, 4 subjects were classified as having CFRD, 3 had IGT and the remaining 4 had NGT. Of these, the 2 subjects who did not complete the second OGTT accounted for 2 of the CFRD group. At the follow up OGTT, 8 of 9 (89%) subjects remained within their respective glucose tolerance status groupings (Figure 1). One subject changed status from IGT during their admission to NGT at follow up. Median (IQR) fasting glucose decreased from 4.9 (4.7–5.2) mmol/L during admission to 4.6 (4.4–5.4) mmol/L at follow up (p = 0.47). Median (IQR) 2 hour glucose for all 9 patients decreased from 11.5 (11–14) mmol/L at admission to 10.0 (9.2–10.7) mmol/L follow up (p = 0.06). Mean change in 2-hour glucose was 1.1 mmol (SD = 0.77). AUC glucose and insulin were both higher during admission (19.1 mmol/L/2hrs and 83.9 µU/ml/2hrs) compared to at follow up (17.2 and 63.4) but the differences did not reach statistical significance (Figure 2). Insulin resistance (mean difference = −0.1(−0.9 to 0.7) (p = 0.74)) and secretion (mean difference −2.2 (−6.3 to 1.9) (p = 0.24)) were not significantly altered during exacerbations.

### Lung Function

Lung function for all 11 patients is shown in Figure 3. FEV_1_ at admission had declined in all patients compared to the best measurement in the previous 6 months. Mean (SD) FEV_1_ decreased from 73.2% (19.2) at baseline to 62.2% (18.9) at admission (t-test, p<0.001). FEV_1_ recovered in the group to 71.2% (22.5) at the time of discharge. This effect was sustained at follow up with a mean (SD) FEV_1_ of 74.7% (20). There was no significant correlation between admission 2-hour glucose levels and recovery of FEV_1_ at follow up (r = −0.05, p = 0.8).

## Discussion

The hypothesis of this present study was that glucose tolerance would be worse during CF pulmonary exacerbations when compared with baseline status. In contrast, the findings of our study do not support this hypothesis. In this present cohort of adolescents with CF, no significant differences in glucose tolerance status were found on OGTT during exacerbations compared with post exacerbation recovery. Only one of nine patients who completed the OGTT component of the study displayed a change in glucose tolerance status between admission and follow up. Furthermore, there was no significant difference in glucose AUC over the 2 hour OGTT between admission and follow up.

This study’s results differ significantly from those of Sc and colleagues, which is the only other similar study to be found in the literature [6]. In their study, 7 of 8 subjects with known NGT had diabetic OGTTs during a pulmonary exacerbation and subsequently retuned to NGT 4 weeks later. In contrast, only one of the 9 subjects who underwent an OGTT in the current study had a change in glucose tolerance status on recovery. The mean difference in 2-hour glucose between exacerbation and recovery in the previous study was 7.6 mmol/L compared to just 1.1 mmol/L in the present study. The reasons for this difference are not immediately apparent. The study designs were similar and the sample sizes were comparable. The definition of a pulmonary exacerbation in the other study was also similar to that of the present study. The patient cohort in this study was younger than that of Sc et al with a mean age of 14.5 years compared to 19.2 years. In addition, FEV_1_ at admission was worse in their group (57.2% versus 62.3%). FEV_1_ at admission in the current study was comparable to another single centre study with a similar age group [11]. Although pancreatic exocrine deficiency is present in the majority of CF patients at or shortly after birth, degradation of the endocrine portion of the gland takes years to become apparent. The prevalence of CFRD increases from about 5% at the age of 10 to about 20% at the age of 20 [12]. It is possible that the younger patients in the current study have more pancreatic endocrine reserve than those in the previous study, allowing them to maintain their glucose tolerance status during pulmonary exacerbations. One major difference between the studies was that we carried out the OGTT using serum samples, which are the gold standard and are more robust than finger stick measurements used in the previous study. Finger sticks give an approximation of blood glucose levels and may vary from true levels by as much as 10% [13]. However, this is unlikely to fully explain the differences between the two studies.

Why then did our present study suggest that glucose tolerance does not differ between acute exacerbations and recovery post exacerbation. One possibility is that the level of stress occurring during the exacerbations was not sufficient to drive hyperglycaemia. In their study examining metabolic and inflammatory response to pulmonary exacerbations in 22 adults with CF, Bell et al found that plasma catecholamines, insulin and glucagon remained unchanged before and after treatment of an exacerbation [14]. Another possible explanation for these findings could be that our patients did not have ‘true’ exacerbations; however our strict inclusion criteria make this unlikely. There are no universally accepted criteria for the diagnosis of CF pulmonary exacerbations [15]. Most criteria are based on subjective measurements such as the physician’s opinion and the patient’s perception of symptoms [16]. Nevertheless, lung function is an objective measurement which is included in most published criteria and a decline in FEV_1_≥10% in the presence of increased symptoms is strongly indicative of an exacerbation.

4 of our 11 patients (36%) admitted during the study were found to have CFRD during admission. This is higher than recently published CFRD data for this age group which reported a prevalence of around 20% [12]. However CFRD is a risk factor for CF exacerbations and therefore patients with CFRD are likely to be over represented in a cohort of patients admitted for pulmonary exacerbations. We cannot say for certain if CFRD was pre-existing in those patients or if the exacerbation ‘unmasked’ latent diabetes. It is possible that the stress associated with an exacerbation may have tipped patients with impaired glucose tolerance over the ‘diabetic’ threshold. Nonetheless, all 4 CFRD patients had a relatively high HbA1c on admission, including 2 above the CFRD diagnostic threshold of 6.5%, suggesting that diabetes preceded the relatively acute deterioration leading to an exacerbation.

Our study found that glucose tolerance status did not change however the 2-hour glucose was lower in the majority of patients following resolution of the exacerbation. The clinical significance of this finding is uncertain. It has been reported that CFRD diagnosed during exacerbations is associated with long term morbidity and mortality [3]. Current international guidelines recommend monitoring of postprandial blood glucose during CF exacerbations [17]. The performance of OGTTs during exacerbations is not currently recommended. The results of this study suggest that it may be reasonable to diagnose CFRD during an exacerbation. Consideration could be given to performing an OGTT towards the end of an exacerbation. It is possible that patients found to be 1–2 mmol/l over the CFRD diagnostic threshold during an exacerbation may return to IGT following recovery. Patients with 2-hour glucose levels higher than this are likely to remain diabetic and should be considered for long-term insulin therapy.

Our sample size was small, although we exceeded the number of patients required to show the same mean difference in 2-hour glucose (7.6 mmol/l) as Sc and colleagues [6]. Furthermore, a retrospective effect size calculation showed that our study had 80% power to detect a smallest average difference in 2-hour glucose of 0.72 mmol/l with a significance level of 0.05 (two-tailed). There are no larger studies in the literature and it is likely that a multicenter study would be necessary to produce a larger data set. It would have been ideal to know the subjects’ glucose tolerance status before entering the study, however the RCH CF unit was not carrying out a comprehensive screening program for diabetes at the time of the study. Instead, a follow up OGTT 4–6 weeks after discharge was used as a measure of the subjects’ baseline status. This design was partially influenced by the study of Sc et al, whose study subjects all returned to baseline status at 4 weeks post discharge [6]. There is objective evidence that our subjects had returned to clinical baseline in that lung function had returned to pre exacerbation levels in all patients at the follow up visit. In addition, all the patients’ symptoms had resolved and physical examinations returned to normal.

In contrast to previous work, the findings of this study show that there is little change in glucose tolerance during CF exacerbations in the majority of patients. In particular, hyperglycaemia found during pulmonary exacerbations cannot be assumed to be transient and at a minimum warrants early follow-up with repeat OGTT following recovery to baseline clinical status. A larger multicenter study is needed to confirm our findings.
